# NEWS – AGENCIES

**DOI:** 10.7189/jogh.07.020202

**Published:** 2017-12

## The Bill and Melinda Gates Foundation

➢In June, Bill Gates gave US$ 4.6 billion of Microsoft shares to the BMGF, followed by a new campaign to combat the spread of malaria. This latest donation reduces his Microsoft holdings from 2.4% to 1.3% (although Microsoft stock accounts for 9% of his overall net worth, and he remains the world’s wealthiest individual, despite donating billions of US$). The anti–malarial campaign focuses on mosquito nets, and is part of the Foundation’s wider efforts to defeat malaria. Donated nets will be distributed by World Vision to families in the Inhambane region of Mozambique, where malaria is still prevalent. The Foundation is also seeking to raise public awareness of malaria, and Mr Gates points out that 429 000 people died from the disease in 2016, although there are “miraculous” falls in malaria deaths since 2000, with numbers halving. (Forbes, 15 August 2017)

➢The state government of Andhra Pradesh (India) is planning to use drone technology in soil testing, and will hold talks with the BMGF to launch a project to assess soil conditions. The BMGF has implemented a similar project in South Africa, and each project aims to assess soil conditions in differing terrains, to determine the most suitable crops, plus crop monitoring. The drones will survey soils across Andhra Pradesh, and will combine the results with data from soil samples to make recommendations. Experience from earlier projects show that this approach can improve agricultural productivity, and the state government will draw upon the guidance of scientists from overseas and India to implement it. The Chief Minister, Mr N Chandrababu Naidu said “it is a unique experiment that forms a part of the government’s endeavor to adapt the latest technologies in the agricultural sector, and it was imperative that the farmers have a reliable database of the diverse soil conditions which have a direct bearing on the yields.” (The Hindu, 29 August 2017)

➢Over the past few years, there has been remarkable progress in reducing infant and child mortality, reducing extreme poverty, ill health, and deaths during childbirth. However, in a report described as a “wake–up call”, the BMGF suggests that progress could be faltering, and that campaigns to eradicate extreme poverty, HIV and malaria are going awry, and this is going unnoticed. This is partly due to demography, as most chaotic countries have high birth rates, and huge fertility gaps have opened between failing states and the rest of the world – and high birth rates place huge strains on working–age populations. Deep poverty is drying up in South Asia, but the falling numbers of people in extreme poverty in sub–Saharan Africa do not outweigh population growth, so overall numbers remain constant. There are risks that HIV infections will rebound, due to complacency over new treatments, and progress in reducing malaria infections may be halted by the rise of drug–resistant parasites. Mr Gates is also concerned that high–income countries may slash their aid expenditure. Despite these real worries, there is room for optimism – better domestic policies could boost well–being without massively increasing expenditure, birth rates may fall faster than expected, and medical breakthroughs could lead to improved treatments. Indeed, the overall value of the report lies more in its estimates of what is at stake if progress stalls – between the most optimistic and pessimistic scenarios lie the lives and well–being of millions of people – and in identifying the largest risks to progress. (Economist, 14 September 2017)

➢Immunocore, a UK–based pharmaceutical company, has received US$ 40 million investment from the BMGF to help develop immunotherapies for infectious diseases, ahead of what is expected to be a much larger funding round. To date, Immunocore has concentrated on applying its T–cell receptor (TCR) technology to treat cancer, but the new investment will extend its reach to fighting infections, starting with HIV and TB. The company’s TCR works by stripping off T–cells receptors, and engineering them to become drugs in their own rights. Dr Chris Karp, the BMGF director of discovery and translational science, said that directing these TCRs against pathogens could treat intractable infections far more effectively than existing drugs. The company emphasises that much more pre–clinical work was needed before the new treatments are ready to test in patients, and if the development goes well, the BMGF would consider further funding, either through grants or equity investment. (Financial Times, 17 September 2017)

➢Africa’s Academy of Science (AAS) has announced an open–access publishing platform – AAS Open Research – the first platform exclusively for African scientists. It will publish articles, research protocols, data sets and codes, mainly within days of submission and before peer review (papers will be indexed after they pass review). The platform is being created by the open–access publisher F1000, and although other open–access publishers already focus on Africa, it is the first to adopt open peer review. It will initially be limited to submissions from AAS fellows and affiliates, as well as researchers funded through programmes affiliated to the Alliance for Accelerating Excellence in Africa; however AAS has a longer–term goal of opening the platform to more researchers. The publication fees (US$ 160 – 1100 per article) will be met by grant funders. Some scientists are concerned that these platforms might hinder African scientists’ career progression if they are not publishing in conventional journals (eg, in South Africa, researchers are rewarded for publishing in journals approved by the country’s Higher Education department). This venture follows a series of open publishing portals launched with F1000 in the past 18 months, including those funded by the Wellcome Trust and the BMGF. (Nature, 15 November 2017)

## The GAVI Alliance

➢Thanks in part to its vast and diverse geography, India faces particular challenges in reaching the poorest and most vulnerable groups with its ambitious vaccination programme, which aims to immunise 156 million people a year. To help overcome these obstacles, India’s government has launched an electronic vaccine intelligence network – eVIN – which is a smart, easy–to–use technology that provides real–time information on vaccine stocks and flows, so that health officials can make quick and informed decisions. It is a cloud–based and mobile application that allows cold–chain handlers to update information on vaccine stocks after each immunisation session. These updates are stored on a cloud server that gives health officials immediate information on stocks and flows, helping them to adjust levels, reduce wastage and empower health workers. eVIN is implemented in collaboration with the UN Development Programme, and its development was financed by GAVI. (Devex, 17 July 2017)

➢Pfizer Inc had made its pneumonia vaccine, Prevanar 13, available at discounted prices to GAVI, where 50 countries are eligible to procure it. However, India has granted a patent to Pfizer, raising concerns that Prevanar 13 would become inaccessible to many in low–income countries. This decision also prevents other companies from making cheaper copies of the vaccine to sell elsewhere, and allows exclusive rights for Pfizer to sell it in India until 2026. The decision by India’s patent court comes at a time of ongoing pressure from the USA for India to tighten its patent laws. Following criticism over the high price of Prevanar 13, Pfizer had reduced the price to NGOs in 2017, but has welcomed the granting of the patent, saying that Prevanar 13 took 2.5 years to develop, and has been available in India since 2010. In 2016, Médecins Sans Frontières filed an objection to Pfizer’s patent application, arguing that a patent would deprive many developing countries of cheaper copies, and is considering its legal options. At least one Indian company is considering a post–grant opposition. (Reuters, 22 August 2017)

➢Cholera is mainly a disease of poverty, preying especially on vulnerable communities in areas with poor sanitation. Each year, 95 000 people die as a results of cholera, many of them children. In 2017, cholera spread at an unprecedented rate in Yemen – where more than 2000 people have died as a result – and there are ongoing outbreaks in Somalia, South Sudan, Haiti and other countries across sub–Saharan Africa and Asia. Cholera is entirely preventable, and the WHO has pledged to end it by 2030. Cholera has to be tackled with a multi–sectoral approach: investments in water, sanitation and hygiene are essential; alongside easy access to treatments such as oral rehydration solutions and intravenous fluids, and the proactive use of oral cholera vaccines. The WHO maintains a global stockpile of cholera vaccines, with support from GAVI, and more than 15 million doses have been distributed to 18 countries since the programme’s inception in 2013. In 2018, the stockpile will increase to 25 million – a huge increase from its original 2 million base. (Irish Examiner, 11 October 2017)

➢Ahead of the meeting of GAVI’s board of directors, Médecins Sans Frontières (MSF) has called on GAVI to ensure the sustainable access to immunisations by putting children’s health at the centre of its funding model, highlighting how nearly 1–in–10 children worldwide do not receive any vaccinations. MSF states that GAVI support is solely assessed on a country’s Gross National Income per capita (GNI), with a current threshold of US$ 1580. Once this threshold is reached, GAVI support is withdrawn over a 5–year period, and the country is expected to increase domestic funding for immunisation, eventually reaching of funding. By the end of 2020, 20 countries will have lost all GAVI funding, 16 countries will have lost funding by the end of 2017, and 8 countries have already lost funding. However, some of the countries scheduled to lose GAVI support have poor or declining immunisation coverage, and the board meeting will consider plans to support their transition. MSF calls on GAVI to strengthen its funding model, by factoring in measures of immunisation coverage, and not rely solely on economic criteria. (ReliefWeb, 28 November 2017)

➢GAVI has agreed US$ 85 million of funding towards the bulk–buying and introduction of typhoid vaccines in low–income countries. GAVI said that it expects the first countries to apply for the vaccine in 2018, and aims to roll it out further in 2019 for children aged over 6 months. Typhoid is a serious – often deadly – disease caused by consuming contaminated food or water, affecting 12–20 million people worldwide each year. 126 000 people died from the disease in 2016, and those who survive become chronic carriers of the disease. Although typhoid is treatable with antibiotics, access to these drugs is limited in low–income countries, and there are increasing prevalence of drug–resistant bacteria. Indeed, GAVI’s chief, Mr Seth Berkeley says that the growing spread of drug resistant strains of typhoid posed a major threat, to which a vaccine could a valuable defence. “Strong coverage through immunisation together with efforts to improve access to clean water and hygiene will play a key role in dramatically reducing the disease,” he said. (Reuters, 30 November 2017)

## The World Bank

➢The World Bank has launched the first pandemic emergency financing instrument, or “pandemic bond”, for epidemics. It will support emergency financing to rapidly fight counteract future health crises, such as the Ebola outbreak in 2014. Mr Michael Burnett of the World Bank, noted that if this instrument had been available in 2014, US$ 100 million could have been mobilised early in the Ebola outbreak. The instrument will offer coverage to all countries eligible for support from the International Development Agency, and payments will depend on the size of disease outbreak, growth rate and the number of countries affected. According to Mr Bennett, it will provide more than US$ 500 million of coverage against pandemics over the next 5 years, against infectious diseases such as pandemic influenza strains, filoviruses, plus others such as Rift Valley Fever and Lassa fever. The World Bank will pay bondholders the equivalent of the insurance premium plus a funding spread, in return for a payout if the bond is triggered, and was oversubscribed by 200% at its launch. Pandemics are the most likely uninsured risk to incur, and the World Bank estimated that the annual global cost of moderately severe to severe pandemics is estimated at approximately US$570 billion – 0.7% of global GDP. (Reuters, 28 June 2017)

➢According to a study from the World Bank, the food produce destroyed by drought would feed 80 million people a year, noting that whilst flooding and storms have an immediate impact on food supplies, droughts are slower–acting. Problems caused by droughts are passed onto the next generation, as women born during droughts have less access to education, have more children and are more likely to suffer from domestic violence. When crop yields fall during droughts, farmers can be forced to cultivate forests – and deforestation can further decrease water supply and exacerbate climate change. The World Bank said many countries affected by drought are overlapped with areas with large food shortages and are classified as fragile, intensifying the need to tackle the problem. It recommends constructing new water storage and management infrastructure, combined with measures to control demand for water, safety nets to help families cope with the economic consequences of drought, and incentivise utility companies to invest and improve their water efficiency. (The Guardian, 24 October 2017)

➢The World Bank defines extreme poverty as living on US$ 1.90/d or less – mostly spent on food, which may be insufficient to ward off hunger or malnourishment. At this level of income, housing will usually be inadequate, and in the absence of free education or health care, insufficient to cover school fees or health expenses. Whilst millions of people in extreme poverty live in low–income countries, more than 50% live in middle–income countries like India, Nigeria and China. However, wealthier countries have higher poverty lines, whilst low–income countries have lower poverty lines. This has led to the World Bank setting “poverty line” figures for middle–income countries: US$ 3.20/d for lower middle–income countries (eg, Egypt and the Phillippines), and US$ 5.50/d for upper middle–income countries (eg, Brazil, Jamaica and South Africa) – and, US$ 21.70/d for high–income countries. These new standards give a benchmark to measure progress in poverty reduction, and for middle–income countries to assess their progress. The UN has pledged to end extreme poverty by 2030, and the poverty lines remind us that although extreme poverty is falling, deep, intractable pockets remain. (NPR, 25 October 2017)

➢According to the International Monetary Fund (IMF), the median level of government debt in sub–Saharan Africa will probably rise to 50% of GDP in 2017, from its current level of 34%. The increase is due to slow economic growth, falling commodity prices, widening fiscal deficits, depreciating currencies, and is increasing pressure on SSA’s financial sector and limiting its potential for stimulating economic growth. The number of low–income countries in debt distress, or facing a high risk of debt distress increased from 7 in 2013 to 12 in 2016, and several countries have had their credit ratings downgraded. “High levels of public debt can be quite harmful,” said the Director of IMF’s Africa Department, Abebe Selassie. “The debt–servicing cost can be a major source of drain of resources that could otherwise be used.” (Bloomberg, 30 October 2017)

➢The International Monetary Fund’s (IMF) latest *World Economic Outlook* predict a year of healthy economic growth in 2018 for the global economy, with low levels of market volatility. In September 2018, central banks may curtail or stop bond purchase programmes which were established to drive down interest rates and provide economic stimulation. For several years, the IMF has reported below–trend economic growth, but now says that “the global upswing in economic growth is strengthening.” The US economy is predicted to grow by 2.5% in 2018, China by 6.4%, Japan by 0.9% and Germany by 1.6%, although the IMF points to “lacklustre” growth in many nations of sub–Saharan Africa, the Middle East and Latin America, and wealthy economies may be affected by wage stagnation. Stronger economic performance is based on confident consumers and companies investing in resources, and is not dependent on a particular region or sector; and it places the world in a better position to deal with the next economic downturn. Policymakers can now start to focus on normalising interest rates and repairing their national finances. Moreover, economies – to date – have been resilient in the face of uncertainty, with South Korea remaining prosperous in the face of threats from North Korea, corporate scandals and warnings from the US President that their free–trade agreement might not survive; and the UK economy continues to growth despite uncertainty over its exit from the European Union. One concern for the global economy as a whole is that recovery has been built on too much debt, which companies will struggle to service when growth eventually weakens. (Bloomberg, 2 November 2017)

## UN AIDS and the Global Fund

➢According to UNAIDS, tuberculosis (TB) is one of the most common causes of death amongst HIV–positive people, causing about 33% of AIDS–related deaths in 2015. Rwanda, which has a reported HIV prevalence rate of 3% – with 6.3% in Kigali – is piloting the early screening for TB amongst people living with HIV in Kigali. This pilot phase will cover about 30% of people infected with TB in Rwanda. Currently, most HIV–positive people only go for screening after symptoms appear, but treatment is more successful if started early. The pilot will be rolled out across Rwanda’s 18 districts, targeting six health centres in each district. Each health centre will have a Peer Educator who will mobilise people living with HIV to attend screening, and screening should target the general public, including prisoners, children aged under 15 and adults aged over 55, who all have higher risks of TB infection. Dr Betru Woldesemayat, the UNAIDS country director for Rwanda said “the initiative to focus on Kigali City will contribute to advancing of the Fast Track agenda in the cities where HIV prevalence is much higher.” He also noted that a strengthened contribution of civil society organisations to finding a combined solution will have a positive impact on the population. (New Times Rwanda, 23 June 2017)

➢The latest report on the HIV pandemic from UNAIDS shows that that the number of HIV–positive people receiving treatment has reached a record 19.5 million – out of a total of 36.5 million people living with HIV. Moreover, AIDS–related deaths have fallen by nearly 50% since their peak of 1.9 million death in 2005. Sub–Saharan Africa, which has been hit particularly hard by the HIV pandemic is showing encouraging progress, with new HIV infections falling by nearly 30% in eastern and southern Africa (with a related 10–year increase in average life expectancy); and Malawi, Mozambique, Uganda and Zimbabwe have cut new infections by 40% or more since 2010. Despite progress in sub–Saharan Africa, the the Middle East/North Africa, and Eastern Europe/Central Asia, AIDS–related deaths have risen by 48% and 38% respectively, mainly because many HIV–positive people lack access to treatment, although the report notes that some countries in those regions who have taken concerted action against HIV have seen better results, eg, Algeria, Morocco and Belarus. Overall, the report found that whilst HIV infections are falling, they are not falling quickly enough to reach global targets, and that that “around 30% of people living with HIV still do not know their HIV status, 17.1 million people living with HIV do not have access to antiretroviral therapy and more than half of all people living with HIV are not virally suppressed.” (Al Jazeera, 20 July 2017)

➢Mr Peter A Sands, a former chief executive of Standard Chartered Bank, was chosen as the new head of the Global Fund. Since its inception 15 years ago, the Global Fund has struggled to raise enough funds to fulfill its mission of combatting HIV, malaria and TB. When it launched in 2002, it was envisaged that the Global Fund would raise and spend at least US$ 8 billion annually, but thanks to funding cutbacks it has struggled to raise US$ 4 billion each year. However, thanks to falls in the prices of generic drugs, the Global Fund has claimed to have saved 22 million lives in the developing world, helping 11 million and 7 million people access HIV and TB drugs respectively. After accusations that it was becoming a swollen bureaucracy and that it was enabling aid recipients to pilfer funds, its most recent chief, Dr Mark R Dybul, has been credited with making it more effective and efficient; and in 2016 it was only 1 of only 3 multilateral agencies to earn top marks for “value for money” from the UK's Department for International Development's score–card. Mr Sands stated that he hopes to have “elimination of the three diseases as epidemics firmly in sight” during his term, which ends in 2022. He also said that it would be “premature” for him to outline any changes he plans to make, and could not name any countries that could act as model aid recipients. (New York Times, 14 November 2017)

➢The AIDS Healthcare Foundation (AHF), the largest non–profit HIV organisation providing HIV care to more than 833 000 people in 39 countries worldwide, has called on the Global Fund to end the use of per capita Gross National Income (GNI) as part of the Global Fund’s grant eligibility criteria. Currently, eligibility is based on a country’s World Bank lending group classification – which is tied to its GNI – and on its HIV prevalence rate as a proxy for disease burden. If a country’s per capita GNI exceeds US$ 3955, it is designated as an upper–middle income country (UMIC) and if it does not have a high burden of disease, it becomes ineligible for support from the Global Fund. Support can be withdrawn even if HIV prevalence is increasing – and the AHF argues that the Fund’s “Transition Readiness Assessment Tool”, which considers the capacity of UMICs to sustain HIV programmes, does not take into account any increases in new HIV infections. Dr Jorge Saaverdra of AHF says “right now, a developing country can be cut off from support even if the rate of new HIV infections is skyrocketing and its epidemic is not under control.” The AHF has been spearheading a campaign to change how the World Bank classifies middle–income countries – currently, a country is classified as middle–income if it has per capita GNI US$ 2.76/d – barely above the International Poverty Line of US$ 1.90/d. Instead, the AHF calls for the World Bank to set the middle–income category at the equivalent of US$ 10/d. This would increase those countries’ access to foreign aid, including HIV drugs and other essential medicines. (InDepth News, 26 November 2017)

➢A UNAIDS report published on the 2017 World AIDS Day shows than more men than women are dying from AIDS, despite more women than men being HIV–positive. This is because overall fewer men are tested for the virus, or have access to treatment. The situation is particularly acute in sub–Saharan Africa, where boys and men living with HIV are 20% less likely than HIV–positive girls and women to know their status – and people who are not receiving treatment are more likely to transmit the virus. Moreover, men are more likely to adhere less strictly to their treatment regime, leading to 58% of AIDS–related deaths occurring amongst men, despite the lower prevalence. The report finds than many men refrain from testing because they fear stigmatisation, and are less likely to visit health care facilities so are less likely to be diagnosed with life–threatening conditions. (Voice of America, 1 December 2017)

## United Nations

➢Following violence against Rohingya Muslims in Myanmar, Nikki Haley, the US ambassador to the UN, has called for countries to suspend weapons provision to Myanmar until the military puts sufficient accountability measures in place. The UN has accused Myanmar's government of ethnic cleansing, following the displacement of hundreds of thousands of people in Rakhine State, and Nikki Haley's statement was the first time that the USA has supported the UN's position. However, China and Russia have both endorsed Myanmar's government, and Myanmar itself rejects the accusations and has denounced rights abuses. Thang Tun, Myanmar's national security adviser, told the UN that there was no ethnic cleansing or genocide, and that the country has invited UN Secretary General Antonio Guterres to visit. To date, the Trump administration has mostly followed the Obama policy of forging closer relationship with Myanmar, partly to counter China's growing influence. Aid groups have urged free access to Rakhine, where more than 500 000 people have fled to Bangladesh, but hundreds of thousands of those remaining are cut off from food, shelter and medical care – since the insurgency attacks in August, the Myanmar government has prevented aid groups and the UN from working in the northern part of Rakhine. Despite the Myanmar Red Cross co–ordinating aid in the state, the International Red Cross fears insufficient aid is reaching people. (Reuters, 28 September 2017)

➢Mark Lowcock, the UN humanitarian chief, told the Security Council that more than 13 million people inside Syria still need humanitarian assistance, and nearly 50% are in “acute need”, having fled their homes due to hostilities and with little access to food, health care and other basic needs. He also said that the number of internally–displaced Syrian people fell from 6.3 million to 6.1 million, although levels of new displacement remain high – 1.8 million people between January and September 2017. Moreover, nearly 3 million people remain in besieged or hard–to–reach areas, where the UN faces “considerable challenges” in meeting humanitarian needs. Mr Lowcock said that progress in de–escalating the conflict had not yet led to increased humanitarian access; indeed, recent airstrikes on Al Mayaldin has left medical facilities “inoperable”, heavy fighting and airstrikes continue to cause civilian deaths and casualties in the province of Deir el–Zour, and fighting in the city of Raqqa had resulted in 436 000 people being displaced. The UK’s UN ambassador, Matthew Rycroft, called the situation in estern Ghouta “atrocious”, and that de–escalation should not mean bombardment. “What we fear is that the de–escalation zone is becoming a starvation zone. So we call on the Syrian regime and their allies to lift the blockade to allow humanitarian aid to get through,” he said. (Voice of America, 30 October 2017)

➢The UN has described the health care available for refugees at a detention centre on Papua New Guinea’s Manus Island has “inadequate”, which 600 refugees are refusing to leave despite essential services being cut off. They are under pressure to relocate to a nearby transit centre. In November a 38–year old refugee with a known heart condition collapsed at a detention centre, and as the island is without ambulance services, a passing car had to be flagged down to take the man to the island’s medical centre. According to sources, the man was not treated at the medical centre as it lacked the necessary equipment, and was sent back to the detention centre. The refugee claims that a drunk security guard prevented him from being visited by staff from the UN refugee agency. Nai Jit Lam, the UN deputy director for the region’s refugees, recently visited the centre, and found that the health care services for refugees had been downgraded. “Many of the refugee population have medical and mental health issues as well which need constant monitoring and constant attention,” he said. Refugees have also reported that the detention centre’s sewer had backed–up, as its pumps were unable to function when the electrical generators were removed. (Radio NZ, 6 November 2017)

➢The UN World Food Programme (WFP) is trialling Distributed Ledger Technology (“blockchain”) to make delivering food assistance faster, more secure, cheaper, and to reach as many people as possible. Under its pilot scheme, Building Blocks, 10 000 refugees in Jordan’s Azraq camp can pay for their food supplies by means of an entitlement recorded on a blockchain–based computing platform. The WFP believes that full implementation of blockchain technology could lead to significant cost savings, potentially millions of US$ each year. Moreover, at November’s Humanitarian Blockchain Summit (organised by the Institute of Humanitarian Affairs, Fordham University and the UN), an initiative to combat child–trafficking was announced, which will use blockchain technology to register undocumented children. (Nasdaq, 17 November 2017)

➢The UN and other international agencies have warned that thousands of Yemeni people could die each day if the Saudi Arabia–led coalition does not lift its blockades on Yemen’s ports. On 6 November, the coalition closed all air, land and sea access to Yemen, following the interception of a missile fired towards Riyadh, saying that the flow of arms from Iran to its Houthi opponents in the the Yemen war had to be stemmed. The UN has appealed for the blockage to be lifted, saying that it could spark the largest famine the world has seen in decades – and 7 million people are already on the brink of famine in Yemen. The UN also reported that the closure of Yemen’s border has halted the delivery of emergency assistance for nearly 280 000 internally–displaced people. In a joint statement, the heads of the World Food Programme, UNICEF and the WHO said that unless all ports are re–opened, Yemen’s 7 million people at risk of famine could grow by 3.2 million. Moreover, at least 1 million children are at risk if a fast–spreading diphtheria outbreak is not halted, and the lives of 400 000 pregnant women and their babies are threatened by a shortage of drugs. The UN refugee agency is also alarmed over the deteriorating humanitarian situation, saying that at a centre for displaced Yemenis in Sanaa “hundreds more people are approaching the facility daily, saying they are no longer able to meet basic needs or afford medical care.” (Hindustan Times, 22 November 2017)

## UNICEF

➢UNICEF has warned that renewed fighting in the Central African Republic (CAR) has led to increasing numbers of violent acts against children, including murders, abductions, rape and recruitment into armed groups. The true number of incidents is likely to be much higher than officially reported figures, because humanitarian access is severely limited in many areas due to fighting. In addition to the many brutalities reported, the intensification of violent conflict has resulted in thousands of children being denied their most basic rights to education and health. It is estimated that 94 000 primary school–children could not take their end–of–term examinations because of school closures. Looting by armed groups has caused the closure of many health centres, stopping essential care and routine immunisations for children. “Children in CAR have suffered disproportionately from the waves of violence that have swept the country over the past three years. Armed groups and parties to the conflict must cease these flagrant violations of children’s rights and make every effort to keep children safe,” said Christine Muhigana, the UNICEF representative for CAR. (Newswire Canada, 18 July 2017)

➢According to UNICEF, more than 700 million women were married as children – and India alone is home to a third of the world’s child brides, putting it amongst countries with the highest prevalence of child marriages in the world. Despite legal restrictions, the practice of young women marring before their 18th birthday – with the authorities often turning a blind eye – still has much support from Indian society. Even though child marriage is more likely to happen in rural than urban areas, a recent study proved that the practice is not mainly a rural occurrence with nearly 1–in–4 girls being married in rural areas in comparison to 1–in–5 girls in city areas. Furthermore, child marriage affects both girls and boys, but the impact on girls is much greater – leading to devastating consequences such as low female literacy rates, exploitation, sexual violence, domestic abuse, and death in childbirth. Ending child marriage requires the engagement of religious and cultural institutions but also the awakening of boys and men’s consciousness in “getting the message out, using social media and school text books, and starting from a young age,” says charity ActionAid. (Reuters, 21 July 2017)

➢Ratul Narain, a graduate from Stanford University, has invented a small bracelet which is fitted to premature babies, and sounds an alarm if the baby’s temperature falls below 36.5°C, prompting intervention and treatment. It is manufactured by Bempu Health in Bengaluru, and has been recognised as one of the 25 best innovations in 2017 by TIME magazine. About 10 000 bracelets have been used world–wide (including Pakistan, Papau New Guinea, Togo and Ghana), via UNICEF initiatives. In India, 1–in–3 newborn babies are of low birthweight, or weight less than 2.5 kg, compared to 1–in–12 in developed countries. Before developing the device, Ratul spent a year in various hospitals to understand neonatal complications. “Among babies with low birth weight, infections can occur at home. Up to 15% of low–weight newborns discharged from government NICU would die at home due to complications like infections and hypothermia. The significant cost of facility care for the baby was, therefore, lost at home. That’s what made me work on a low–cost solution,” said Ratul. (Times of India, 27 November 2017)

➢UNICEF has warned that there is little progress in halting HIV amongst children, with 18 children every hour being infected with the virus in 2016. If this trend continues, there will be 3.5 million new cases of HIV infection amongst adolescents by 2030. Worldwide, nearly 37 million people are living with HIV, and this includes 2.1 million adolescents – a 30% increase from 2005. In 2016, 55 000 adolescents and 120 000 children died from AIDS–related causes. HIV–positive children aged under 4 years have the highest risk of dying from AIDS–related causes. UNICEF states that that nearly all the adolescent deaths were in sub–Saharan Africa, and that more girls than boys are infected. The testing and treatment of babies is also falling behind, with less than 50% of HIV–exposed infants being testing in the first 2 months of life. UNICEF confirmed progress on arresting mother–to–child transmission of HIV, with 2 million new infections being averted since 2000, but said that progress was slowing. UNICEF calls for an array of actions, including the treatment of all infected children, and prioritising interventions for adolescent girls in sub–Saharan Africa. “It is unacceptable that we continue to see so many children dying from AIDS and so little progress made to protect adolescents from new HIV infections,” said Dr Chewe Luo, head of HIV at UNICEF. (Voice of America, 27 November 2017)

➢Libya’s Man–Made River (MMR) authority, the international community and UNICEF have been working together to ensure the functioning of Libya’s water system. This is following attacks on the Hasawanah reservoir (which serves Tripoli) by a Brak Al–Sharti–based group who are demanding the release of its leader, Mabrouk Ahnish. Mr Anhish is being held in prison by Rada forces. This is the second time in two months that the group have closed the water system. However, the water system has now been reopened and the reservoir is slowly refilling – according to the MMR, it will take 3 more days for water levels to return to normal. The closure of the water system meant that 2 million Libyan people, including 600 000 children in Tripoli and its surrounding areas, were left without running water. This forced people to resort to potentially unsafe or contaminated water, increasing the risks of disease outbreak and adding to the suffering of Libyan children. “Access to water is a fundamental human right and international humanitarian law protects civilian infrastructure and the rights of civilians to access basic services,” said Mr Abdel–Rahman Ghandour, the UNICEF special representative for Libya. (Libya Herald, 3 December 2017)

## World Health Organization (WHO)

➢According to the WHO, expanded access to Community Health Workers – a core component of primary health care – could prevent up to 3 million deaths per year. This could result in an economic rate of return on investment of 10–to–1 in sub–Saharan Africa, but primary health care remains the most underfunded, and commonly overlooked, area of health care. Primary health care also delivers vital maternal objections that would remain unmet in areas with limited or no access to mid–level care. Toyin Ojora Saraki, founder of the Well–being Foundation Africa, argues that the absence of established institutions and infrastructure, combined with chronic funding shortfalls, means that primary health care is often the only form of care in many developing countries. She argued that it can reduce inequalities, thanks to the grass–roots provision of care, and investing in primary health care would have a greater impact on the world’s poorest people, compared to investing in mid–level care. She highlights barriers to extending primary health care, including lack of resources and government commitment to driving ti forward, and calls for greater focus on improving the quality of primary health care services around the world, and that without effective monitoring systems, there is little incentive to make improvements. (Huffington Post, 9 August 2017)

➢Following global condemnation, WHO’s Director General, Tedros Ghebreyesus has rescinded the Zimbabwean President, Mr Robert Mugabe’s, appointment as a WHO “goodwill ambassador”. According to the campaigning group, Human Rights Watch, the appointment “embarrasses” the WHO and its leadership, and the US State Department said that it “clearly contradicts the United Nations ideals of respect for human rights and human dignity”. The *New York Times* highlighted in 2009 how Mr Mugabe’s regime had wrecked Zimbabwe’s health care system, leading to cholera and the spread of other diseases. Indeed, the international response centred on one point – can some–one be a goodwill ambassador if they are widely regarded as a violent, tyrannical despot? Zimbabwe’s government confirmed that it respected the WHO’s decision to withdraw Mr Mugabe’s appointment, but noted that Mr Mugabe has already contributed hugely to the world’s fight against non–communicable diseases. (Washington Post, 22 October 2017)

➢According to a report from Results UK, an advocacy organisation that works to influence political decisions on health, education and economic opportunity, officials are overlooking a new, potential problem as the eradication of polio grows nearer. The Global Polio Eradication Initiative and the US$1 billion it channels each year into the WHO is already being wound–down – funding is due to be halved by 2019, and will cease thereafter, except in countries that are still battling polio or at high risk of its return. This could severely undermine low–income countries’ efforts to continue to vaccinate against polio and other diseases, such as measles and rotavirus, plus damaging the surveillance network needed to ensure that the disease is truly gone – 70% of surveillance funding comes from this initiative. The end of the polio programme could place substantial financial pressure on the WHO, which receives 25% of its funding from the polio campaign, and the report also raises the alarm over the state of planning for the end of the polio eradication effort. The WHO raised its “great concern” on its reliance on this funding at the May World Health Assembly, and the risk to its capacity to deliver key programme areas and to maintain essential ongoing functions. The polio vaccine is currently administered orally, but it will be replaced by an injectable vaccine, and the WHO recommends the use of injectable vaccines for at least a decade after eradication. However, injectable vaccines must be given by trained health care workers, and requires different delivery models – which must be more conducive to the delivery of other childhood vaccines. However, the winding down of the polio eradication effort could give an opportunity to refocus efforts on ensuring that countries have the systems and capacity to vaccinate all children with the 11 WHO–recommended vaccines, but without the necessary planning, the opportunity could be lost. (STAT, 13 November 2017)

➢The WHO and member countries has a target of cutting tuberculosis deaths by 95%, a 90% reduction in new cases, and to “ensure that no family is burdened with catastrophic expenses due to TB.” However, Médecins Sans Frontières (MSF) has expressed grave concerns over South Africa’s ability to reach these targets. Tackling TB in South Africa is a global concern, as its high HIV incidence predisposes people to TB, and its increasing rate of drug–resistant TB. South Africa has seen falling death rates from TB since scaling–up access to drugs effective against drug–resistant TB – in particular, bedaquiline – although there is general agreement that drug treatments are not enough to tackle the problem of TB. Dr Fareed Abdullah, director for AIDS and TB Research at the Medical Research Council, calls for improved diagnostic tests, agreeing that 100–150 000 TB cases are missed each year in South Africa, and 4 million globally. Improved screening of each patient’s contacts is also needed, and South Africa has missed many opportunities for prevention, eg, by not administering drugs to prevent infection from developing into active illness in children. His main hope for combatting TB lies with a new vaccine, and that if the world is to end TB by 2035 “we require new tools and investments to do it.” (Daily Maverick, 20 November 2017)

